# Spatial exploration of non-resilience to food insecurity, its association with COVID-19 and household coping strategies in East Gojjam districts, Northwest Ethiopia, 2020

**DOI:** 10.1038/s41598-022-19963-2

**Published:** 2022-09-15

**Authors:** Ayenew Negesse, Wubetu Woyraw, Habtamu Temesgen, Yohannes Teka, Lieltwork Yismaw, Tadesse Yirga Akalu, Yikeber Argachew Deml, Bickes Wube Sume, Yilkal Negesse, Tesfahun Taddege, Wassie Dessie Kidie, Abraham Teym, Biachew Asmare, Yidersal Hune, Dawit Damte, Temesgen Getaneh, Tsige Gebre, Bayu Tilahun, Aemero Tenagne, Eniyew Tegegne, Molla Yigzaw Birhanu, Habitamu Mekonen, Mulu Shiferaw, Woldeteklehaymanot Kassahun, Beruk Berhanu Desalegn

**Affiliations:** 1 Department of Human Nutrition, Health Science College, Debre Markos University, Debre Markos, Ethiopia; 2 Department of Public Health, Health Science College, Debre Markos University, Debre Markos, Ethiopia; 3 Department of Pediatrics and Child Health, Health Science College, Debre Markos University, Debre Markos, Ethiopia; 4 Department of Biomedical Sciences, School of Medicine, Debre Markos University, Debre Markos, Ethiopia; 5 Department of Epidemiology and Biostatistics, Health Science College, Mizan Tepi University, Tepi, Ethiopia; 6 Amhara Public Health Institute, Bahir Dar, Amhara Regional State Ethiopia; 7 Department of Hydraulic and Water Resources Engineering, Institute of Technology, Debre Markos University, Debre Markos, Ethiopia; 8 Department of Environmental Health, Health Science College, Debre Markos University, Debre Markos, Ethiopia; 9 Love in Action Ethiopia (LIAE), Addis Ababa, Ethiopia; 10 Department of Midwifery, Health Science College, Debre Markos University, Debre Markos, Ethiopia; 11 Departement of Health Informatics, Health Science College, Debre Markos University, Debre Markos, Ethiopia; 12 SafeHands “Labour at the Last Mile”, London, UK; 13 Department of Biomedical Sciences, School of Nursing, College of Health Sciences, Woldia University, Woldia, Ethiopia; 14 Department of Medical Laboratory Sciences, College of Health Sciences, Woldia University, Woldia, Ethiopia; 15 School of Nutrition, Food Science and Technology, Hawassa University, Hawassa, Ethiopia

**Keywords:** Diseases, Health care, Risk factors

## Abstract

The coronavirus disease-2019 (COVID-19) pandemic has posed a significant multifaceted threat to the global community. Ethiopia, as a Sub-Saharan African country, is suffering from chronic food insecurity, and the emergence of such a pandemic will exacerbate the situation. As a result, this study investigated the spatial variation of non-resilience to food insecurity, its relationship with COVID-19, and household coping strategies to become resilient in the long run among households in the East Gojjam Zone of Northwest Ethiopia. From September 22 to December 24, 2020, an agro-ecological-based cross-sectional study of 3532 households was conducted to assess the spatial distribution and associated factors of non-resilience to household food insecurity. The enumeration areas (EAs) and households were chosen using a multistage sampling technique. Data were gathered using a semi-structured questionnaire and checklist using an Android device loaded with an Open Data Kit (ODK) template. Binary logistic regression was used to identify the specific factors associated with household non-resilience to food insecurity. A thematic analysis was conducted to investigate the opportunities and challenges of resilience for household food insecurity. Nearly two-thirds (62.5%) of the households were farmers, 67.9% lived in rural areas, and nearly three-quarters (73.8%) earned less than or equal to ETB 2100 per month. Males headed more than four-fifths of the households (81.7%). We found that nearly two-thirds of the households (60.02%), 95% CI 58.40, 61.64) were food insecure. After bivariate logistic regression, we found that households who were divorced (AOR = 2.54 (1.65, 3.87)), daily laborers (AOR = 2.37 (1.15, 4.87)), government employees (AOR = 2.06 (1.05, 4.05)), residents of highland and hot areas (AOR = 11.5 (5.37, 16.77)) and lowland areas (AOR = 1.35 (1.02, 3.15)) were frustrated by COVID-19 (AOR = 1.23 (1.02, 1.50)) and price inflation (1.89 (AOR = 1.42, 2.56))) were at higher odds of being non-resilient to household food insecurity at a 95% confidence level. Geospatial hot spot analysis revealed that Kurar kebele (the lowest government administrative unit) in Dejen District and Debre Markos town were the red-hotspot areas of household non-resilience to food insecurity. Less than a quarter of the households attempted to cope with food insecurity by adjusting their food consumption, while more than 60% of the households chose none of the coping strategies tested. According to the thematic analysis, the degree of poverty (lack of asset ownership), the COVID-19 pandemic, farm decreased variety, and low crop productivity were identified as challenges to coping with the hardship of resilience to food insecurity. During the COVID-19 pandemic and public emergency, the proportion of households that were unprepared for food insecurity reached its peak. It was recognized that a segment of the population with low economic capacity was more vulnerable to food insecurity and less resilient. Tough developmental gains will be undermined in this case. As a result, each responsible body and stakeholder should develop and implement solid corrective plans for the local context.

## Introduction

The coronavirus disease 2019 (COVID-19) caused by SARS-CoV-2 is now a global public health emergency^1^. Remarkably, this pandemic rapidly affects all segments of the global population, including Ethiopia^2^. However, the case fatality rate varies regardless of age, gender, ethnicity, socioeconomic status, or health status^3^. COVID-19 has now become a critical issue, exposing numerous associated problems such as panic and public anxiety throughout the population^4^, livelihood insecurity^5^, particularly food insecurity, and food crisis^6^. Food insecurity, in particular, is explained by the quantity and quality of available food, uncertainty about food accessibility, and personal experiences with going hungry during a public emergency^7^. This issue may be experienced at the individual or household level with a lack of food for consumption, allocation, and the physiological sensation of hunger^8^. It can be measured using the Food and Agriculture Organization's (FAO) method for estimating calories available per capita at the national, regional, and district levels through the use of food balance sheets (the net value of total food produced + food imported + food aids-food exported -foods expended for extra consumption), household income and expenditure surveys, individual dietary intake, anthropometry, and experience-based food insecurity measurement scales^9^. However, the commonly used measurement tools of food insecurity in Ethiopia are the Household Food Insecurity Access Scale (HFIAS)^10^ and the Household Hunger Scale (HHS)^11^. The Household Food Insecurity Access Scale (HFIAS) is the most commonly used food insecurity measurement tool in Ethiopia. It can also be measured using the United Nations FAO Resilience Index Measurement and Assessment II (RIMA-II)^12^. According to a 2017 UN World Food Program report, more than one hundred million people experienced severe food insecurity^13^. There were also nearly one billion people who went hungry, despite the fact that enough food was produced to feed the world's population^14^. Household food insecurity is more prevalent in Sub-Saharan African (SSA) countries. As per the FAO report, food insecurity affected more than one out of every ten households in SSA countries in 2016^15^. In 2017, however, more than half a billion people required emergency food assistance^16^.

Food insecurity has been a major issue in different parts of Ethiopia for over a decade, resulting in a high morbidity and mortality rate^17–19^. From a survey conducted among rural households in Ethiopia between 2018 and 2019, more than half of the households were food insecure^19^. In most of the studies performed earlier, household non-resilience to food insecurity was used to measure the capacity for resilience to food insecurity. Multiple household food insecurity resilience studies from different parts of Ethiopia have shown a very large proportion of the households (more than half up to three-fourth of the households) were non-resilient^20–22^.

Prior to the emergence of COVID-19, more than two-thirds of households in the East Gojjam districts experienced food insecurity, which was more pronounced in the low lands of the Abay valleys and in hilly and mountainous areas^23^. According to recent studies from the neighboring zone of East Gojjam, only one-fifth of the households are resilient to the issue of food insecurity^24^. This is an early warning sign of how difficult household resilience is even in a normal environment, and how it may be even more complicated in an era of public emergency and pandemic disease, such as COVID-19^25^. A multi-country cross-sectional survey conducted across nine African countries (Chad, Djibouti, Ethiopia, Kenya, Malawi, Mali, Nigeria, South Africa, and Uganda) revealed a dramatic increase in non-resilience to food insecurity as a result of this COVID-19 pandemic and public emergency^26^. Another similar study in Ethiopia, Malawi, Nigeria, and Uganda also found that 77% of the population lives in households were lost income as a result of this pandemic and public emergency^27^. Despite the lack of reporting on spatial distribution, one pocket study in Ethiopia also found that nearly 90% of households experienced non-resilience to food insecurity immediately after the occurrence of this pandemic disease and public emergency^5^, which was significantly higher prior to the occurrence of this pandemic and public emergency disease^28–37^. After COVID-19 was declared by WHO as a global health emergency and a pandemic disease, the efforts of humanitarian and food security organizations seeking to reverse food insecurity, resilience, and coping strategies were undermined^25^.

While the government ordered people to stay at home due to the COVID-19 pandemic, the issue of Ethiopia's non-resilience to food insecurity became an immediate concern. As a result, household non-resilience to food insecurity in the COVID-19 era may differ significantly from previous studies^23,24^. Despite, the spatial distribution of COVID-19 incidence rates was high in the urban compared to rural at the beginning of the epidemic, the intensity of the epidemic had also shifted to a rapid surge in rural areas too^38^. As a result of the COVID-19 lockdown, many people around the world, including Ethiopia, have lost their jobs, raising concerns about food availability, distribution, access, utilization, and supply chains^39,40^. In the Ethiopian context, including our study sites, the pandemic has eroded hard-earned development gains and threatens household resilience to food insecurity, while also affecting international organizations working to improve household non-resilience to food insecurity^41,42^. As a result, using gaps from international evidences and lessons from previous pandemic diseases, the authors were keen to investigate the spatial distribution of non-resilience to food insecurity, associated factors, challenges and opportunities as well as the coping strategies of it by taking into account agro-ecological data, soil types, and population residence (urban versus rural data). As a result, this study will be critical for both the regional government of Ethiopia and humanitarian organizations that are interested in providing project-based livelihood support and developing mechanisms for long-term mitigating strategies for household food insecurity that occurred during the COVID-19 pandemic and public emergency.

## Methods and materials

### Study area and period

This study was conducted in the East Gojjam Zone of Amhara National Regional State (ANRS), Ethiopia, from September 22 to December 24, 2020. The East Gojjam Zone, one of ANRS's twelve zones and three city administrations, is located in the northwest of Ethiopia and the southwest of ANRS. Debre Markos serves as the administrative center and is located approximately 299 km and 264 km from the capitals of Addis Ababa and Bahir Dar, respectively. East Gojjam is divided into 19 districts and three town administrations. The zone has a population of more than 2,496,325 people, with 1,221,255 males and 1,275,070 females. The population is spread across six agro-ecological zones, ranging from hilly and mountainous terrain to the lowlands of the Abay gorge^43^. Residents of these two extremes are highly likely to face food insecurity^23^, particularly emergent food insecurity and food crisis during the COVID-19 pandemic^44^.

### Study design and population

A cross-sectional community-based study with a concurrent mixed methods design was used. This study's source population consisted of all households in the East Gojjam zone. All households in the East Gojjam zone's selected districts and town administrations were included in the study population. Community leaders, religious fathers, well-known community elders, and responsible bodies from the food and nutrition security department, disaster prevention and preparedness department, trade and marketing management department, social security and welfare department, and child and women department participated to assess government preparedness, response, and risk mitigation options, as well as anticipated challenges and opportunities.

### Eligibility criteria

This study included all households with a permanent address in the enumeration areas that were chosen (EAs). Households that had been shut and whose residents were not available after three attempts of visit, as well as household heads who were unable to participate in this study, were planned to be excluded.

### Sample size determination

This study considered a sample size calculation for survey study by using the following formula^45^:

$$N=\frac{\left[4(r)(1-r)(d)(1.1)\right]}{\left[{\left(0.12r\right)}^{2}\left(p\right)(h)\right]}$$

$$N =\frac{\left[4(0.79)(1-0.21)(2)(1.1)\right]}{\left[{\left(0.12*0.79\right)}^{2}\left(0.01\right)(4.6)\right]}$$

$$ {\text{N}} = {\text{ 3532 households}} $$

where; N is the required total sample size, 4 is a factor to achieve the 95% level of confidence, r is the predicted or anticipated prevalence (coverage rate) of un-resilence among households(0.79)^24^, 1.1 is the factor necessary to raise the sample size by 10% for non-response rate, d is design effect = 2, 0.12r is the margin of error to be tolerated at the 95% level of confidence, defined as 12% of r (12% thus represents the relative sampling error of r), p is the proportion of the total households in the east Gojjam zone compared with the national estimates (P = 0.01), h is the average household size = of 4.6 individuals per household (EDHS, 2016).

Taking into account the aforementioned parameters as well as the %age of households that were not resilient to food insecurity (79%)^24^, the final sample size is 3532 households, which accounts for 505 households in each selected district and town administration.

### Sampling procedures

#### For quantitative

**Figure 1:**
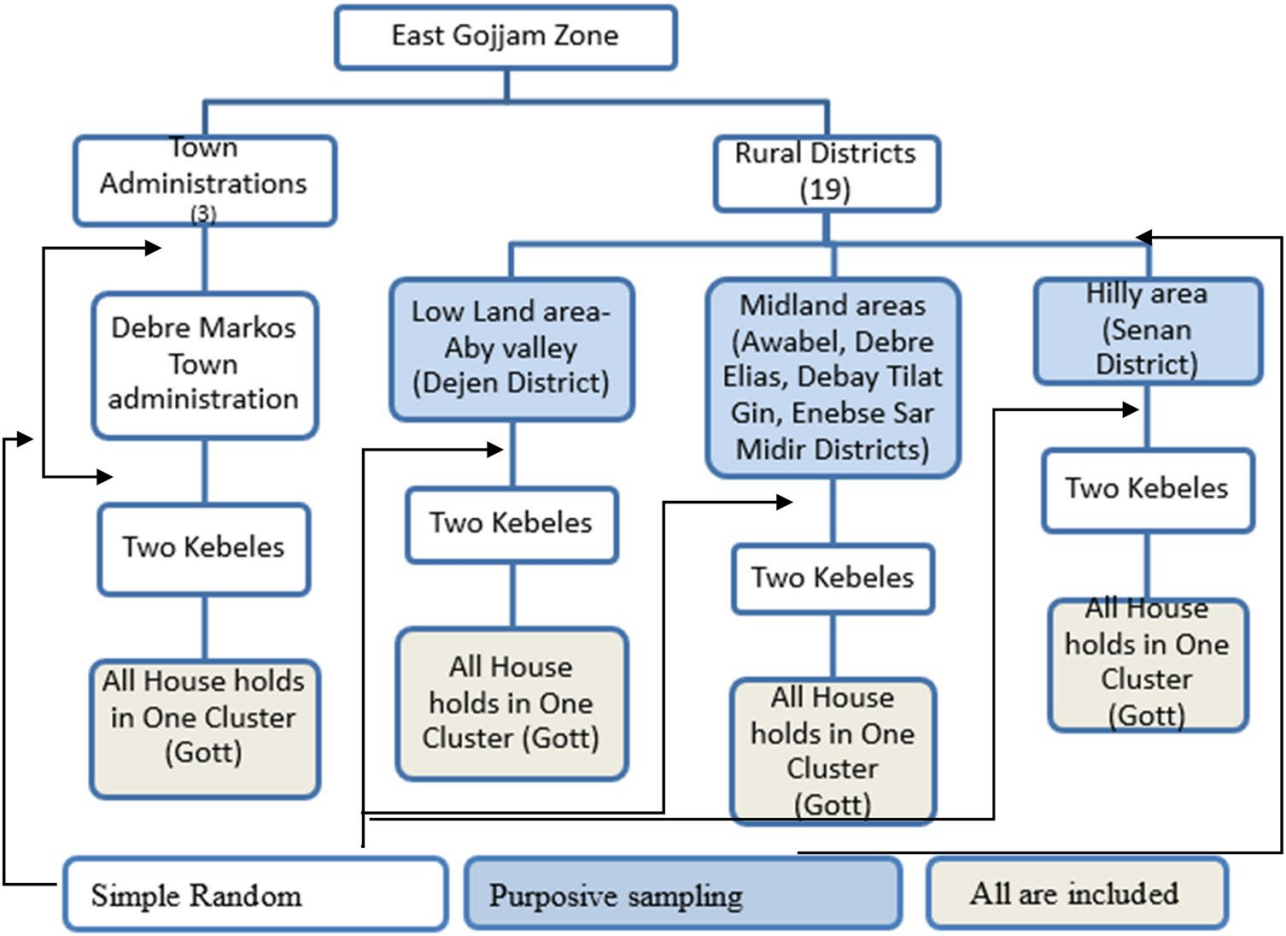
Schematic presentation of the sampling procedure.

Using a random sampling technique, one of the three town administrations was chosen for this study. Six districts were chosen using a purposive sampling technique from the 19 districts of the East Gojjam zone based on agro-ecological and weather conditions. To select clusters/gotts (subdivisions in a Kebele), multistage sampling technique was used. Two kebeles (the lowest government administrative unit) from each of the selected town administrations and two kebeles from each of the selected districts were chosen using a simple random sampling technique. Then, at random, one cluster was chosen from each kebele. Finally, the study included all eligible households in the chosen cluster (Fig. 1).

#### For qualitative

A criterion-based purposive sampling technique was used to recruit key informants and in-depth interviewees. Individuals with good awareness and experience about the issue of household non-resilience to food insecurity, individuals with experience and working on disaster preparedness and management, individuals with good awareness of the community environment and representative of the localities, and individuals on the COVID-19 steering committee were considered for the interview. Based on this, each responsible body at the zonal level was interviewed for the qualitative study, including the Food and Nutrition Security department of East Gojjam Zone, Disaster Prevention and Preparedness department, Trade and Market Management department, Social Security and Welfare department, Agricultural and Natural Resource Management department, Child and Women department, and COVID-19 steering committee. Whereas seven key informant interviews with agricultural extension workers and 36 in-depth interviews (8 community leaders, 6 religious’ leaders, 10 elders, and 12 selected individuals from households who had good awareness about non-resilience) were interviewed.

### Variables of the study

#### Dependent variable

- Households Resilience from emergent food insecurity at the time of shock exposure (COVID-19).

#### Independent variables

- *Socio-demographic and economic characteristics:* age, educational status (maternal, paternal), marital status of the head, number of children, family size, occupation of the head, household assets, monthly expenditure, sex of household head;
- *Agricultural extension service-related factors*: Agro-ecological zone
- *Environments that influence food insecurity beyond their household capacity*: Shock exposure (the COVID-19 pandemic), price inflation (of basic food commodities for consumption), and unwanted weather changes.

### Measurement of the dependent variable

#### Household resilience for food insecurity during the COVID-19 pandemic

The Resilience Index Measurement and Analysis II (RIMA-II) validated by the United Nations Food and Agricultural Organization (FAO) was used to calculate the resilience index score^12^. To calculate the unidimensional and weighted resilience indicators, as well as the resilience index score, Principal Component Analysis was used. Then, using the following formula, households were classified as resilient or non-resilient based on the mean value of the resilience score:

$$ \begin{aligned} {\text{Resilience to food insecurity}} & =  0.{14}0{9}*{\text{ pc1 }} + 0.0{822}*{\text{ pc2 }} + 0.0{762}*{\text{ pc3 }} \hfill \\ & \quad + 0.0{596}*{\text{ pc4 }} + 0.0{444}*{\text{ pc5 }} + 0.0{4}0{3}*{\text{ pc6 }} + 0.{388}*{\text{ pc7 }} \hfill \\ & \quad + 0.0{34}0*{\text{ pc8 }} + 0.{335}*{\text{ pc9 }} + 0.0{323}*{\text{ pc1}}0 \, \hfill \\ & \quad + 0.0{32}0 \, *{\text{pc11 }} + 0.0{315}*{\text{ pc12}} \hfill \\ \end{aligned} $$

From the above formula, households with a factor score ≥ 0 are considered resilient to food insecurity, whereas households with a factor score < 0 are considered non-resilient to food insecurity.

### Data collectors, collection tool and collection process

A semi-structured questionnaire was developed, and data were collected through face-to-face interviews using an Android device loaded with open data kit (ODK) forms pretested and adapted from FAO and previous studies. Socioeconomic and demographic characteristics, agricultural extension service-related factors, essential service-related factors, and exogenous factors influencing household non-resilience and food insecurity coping strategies were collected from one of the household families who were ≥ 18 years old and had detailed information about their households. A data collection checklist was also used to collect secondary data on crop production and livestock availability, total import and export exchange in the study year, amount of food stocked-in in case of emergency (COVID-19 pandemic), and market price for basic commodities in each respective district, geographic coordinates, and agro-ecological classification of the study areas. The key informant and in-depth interview guides were designed to evaluate government preparedness, response and risk mitigation options, and anticipated challenges and opportunities. It was gathered through a face-to-face interview by 14 graduating human nutrition and food science students (two for each district and town administration) under the close supervision of 7 supervisors (one for each district and town administration). The interviews lasted no longer than 40 min.

### Quality assurance mechanism

The questionnaire and interview guides were pretested with selected representative groups and professional experts to ensure their appropriateness and clarity. To reduce data entry errors, electronic data collection technique was used. Data collectors received orientation and pre-collection training. The collected data were reviewed and verified for accuracy. Independent experts performed the key informant and in-depth interview translation and transcription. The transcribed data, interpretations, and conclusions were returned to the participants in order for them to correct errors and challenge what they perceived to be incorrect interpretations.

### Data management and analysis

The ODK platform data were exported to a Microsoft Excel spreadsheet and then imported into STATA Version 15 for further statistical analysis. ArcGIS version 10.4 was used to investigate spatial patterns and identify hotspot areas of non-resilient food insecurity. To investigate the spatial pattern of non-resilient food security across the entire study area, a global spatial autocorrelation (GSA) analysis was performed using the Global Moran's I statistic. Moran's I near 1, 0, − 1 indicates that the spatial distribution of resilience to food insecurity is clustered, randomly distributed, and dispersed, respectively-resilience. The Getis-Ord Gi* statistic was used in GSA analysis to detect local clusters in the presence of clustering. The findings were presented in the form of text, tables, and graphs. For descriptive data, proportion and mean were employed. To identify the factors associated with resilience to food insecurity, a binary logistic regression model was fitted. At a P-value of 0.05, the adjusted odds ratio (AOR) was used to assess the strength of the association. ATLASTi7 for qualitative data coding was used to prepare the data for thematic analysis in the qualitative aspect.

### Ethics approval and consent to participate

Debre Markos University's Ethical Review Committee granted ethical approval with protocol number of HSC/R/C/Ser/PG/Co/132/12/12. A letter of support was obtained from the East Gojjam zone administration and each district. At the time of data collection, each study participant provided oral informed consent. Information confidentiality was maintained by encoding study participants' identities. All methods were performed in accordance with the relevant guidelines and regulations.

## Results

### Socio-demographic and economic characteristics

This study had a 100% response rate. More than half of the study participants (52.7%) were females, 62.2% were married, and 67.9% lived in rural areas. Farmers made up nearly one-third (62.5%) of the study participants. Almost three-fourths (73.8%) of the households earned less than or equal to 2100 Ethiopian Birr per month (Table 1).

**Table 1:** Socio-demographic and economic characteristics of the study participants.

Variables	Characteristics	No	%
Sex	Female	1860	52.7
	Male	1672	47.3
Marital status	Divorced	275	7.8
	Married	2196	62.2
	Single	795	22.5
	Widowed	266	7.5
Residence	Rural	2398	67.9
	Urban	1134	32.1
Paternal education	Cannot read and write	1561	44.2
	Can read and write	756	21.4
	College and above	471	13.3
	Primary (grades 1–8)	477	13.5
	Secondary (grades 9–12)	267	7.6
Maternal education	Cannot read and write	2169	61.4
	Can read and write	559	15.8
	College and above	246	7.0
	Primary (grades 1–8)	340	9.6
	Secondary (grades 9–12)	218	6.2
Occupation of the household head	Daily laborer	145	4.1
	Farmer	2208	62.5
	Government employee	402	11.4
	Housewife	167	4.7
	Merchant	610	17.3
Family size	< Five	2839	80.4
	≥ Five	693	19.6
Under-five children	≤ Two	3505	99.2
	> Two	27	0.8
Monthly income	≤ 2100	2605	73.8
	> 2100	927	26.2

### Topographic, climatic and housing characteristics

Simane Belay et al. conducted scientific research on the overall topographic and climatic conditions of our study area^43^. Based on his agro-ecological classification of our study area, our study found that 77.7% of the studied households lived in the Woyna-Dega climatic zone^43^and 81.7% of the households were led by males. With regard to household ownership, 87.5% of the households are living within their own houses. Nearly three-fourths (73.8%) of the households were expending less than 2100 birr per month (Table 2).

**Table 2:** Topographic, climatic, and housing characteristics.

Variables	Characteristics	No	%
Agroecology	Highland and cold area	234	6.6
	Low land area	126	3.6
	High land and hot area	978	27.7
	Mid land area	2194	62.1
Climate	Dega	234	6.6
	Kolla	552	15.6
	Woina-daga	2746	77.7
House ownership	Kebele's rent	59	1.6
	Own house	3091	87.5
	Private rent	343	9.7
	A relative house without rent	39	1.1
Household head	Female	646	18.3
	Male	2886	81.7
Food expense /month	< 2100	2605	73.8
	≥ 2100	927	26.2

### Principal component analysis and dimension reduction

According to the Analytical Hierarchy Process (AHP)^46^, the main variable that explains 54.9% of the resilience is the status of household assets, which has a Principal Eigenvalue of 5.179 (Table 3).

**Table 3:**
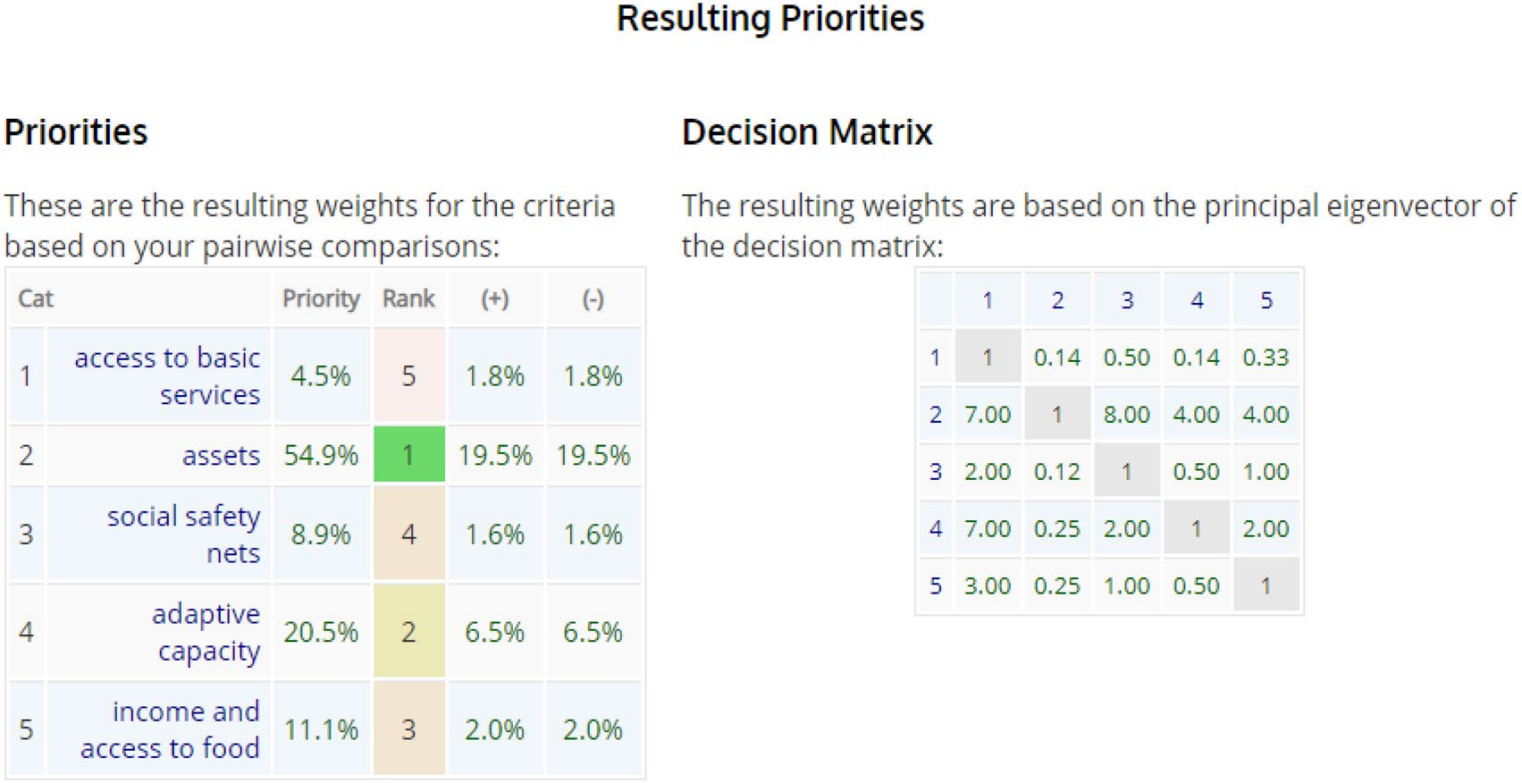
Analytical hierarchy process (AHP) which shows priority matrix.

**Figure 2:**
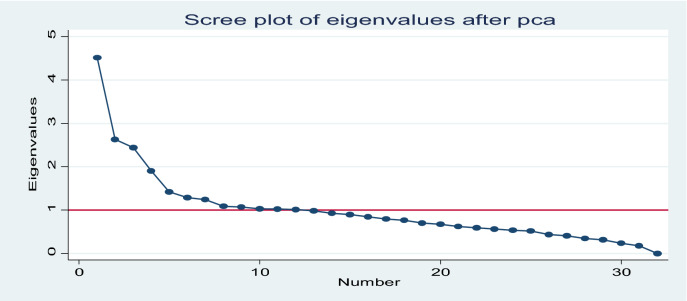
Scree plot showing retained variables after Principal component analysis (PCA).

From the scree plot, 12 variables whose eigenvalues greater than or equal to 1 were retained (Fig. 2).

### Resilience to household food insecurity

Of the total number of households, 2120 (60.02%) were food insecure (95% CI: 58.40, 61.64). More than half of married people (60.6%) were not resilient to food insecurity. During the COVID-19 pandemic and public emergency, the prevalence of household non-resilience to food insecurity was nearly identical in urban (49%) and rural (51%) areas. About 46% of mothers who could not read or write were vulnerable to food insecurity. Of the total households, 87.9% of those with a family size of less than five were also vulnerable to food insecurity. According to the agro-ecological conditions, 38.1% of the non-resilient households for household food insecurity were from the midland with red soils. The Woyna-Dega climatic zones were home to 76.7% households. Respondents reported that COVID-19 was responsible for 52.7% of the non-resilience of household food insecurity and price inflation was responsible for 74.1% (Table 4).

**Table 4:** Prevalence of non-resilience to food insecurity during at this time of COVID-19 pandemic and Public Emergency.

Variables	Characteristics	Resilience status			
		Resilient		Un-resilient	
		No	%	No	%
Marital status	Divorced	70	25.45	205	74.55
	Married	912	41.53	1284	58.47
	Single	316	39.75	479	60.25
	Widowed	114	42.86	152	57.14
Residence	Rural	1316	54.88	1082	45.12
	Urban	96	8.47	1038	91.53
Paternal education	Cannot read and write	893	57.21	668	42.79
	Can read and write	378	50.00	378	50.00
	College and above	42	8.92	429	91.08
	Primary (grades 1–8)	69	14.47	408	85.53
	Secondary (grades 9–12)	30	11.24	237	88.76
Maternal education	Cannot read and write	1193	55.0	976	45.0
	Can read and write	131	23.4	428	76.6
	College and above	12	4.9	234	95.1
	Primary (grades 1–8)	55	16.2	285	83.8
	Secondary (grades 9–12)	21	9.6	197	90.4
Occupation	Daily labourer	12	8.3	133	91.7
	Farmer	1288	58.3	920	41.7
	Government employee	27	6.7	375	93.3
	Housewife	17	10.2	150	89.8
	Merchant	68	11.1	542	88.9
Family size	< Five	976	34.4	1863	65.6
	≥ Five	436	62.9	257	37.1
Monthly income	≤ 2100	944	36.2	1661	63.8
	> 2100	468	50.5	459	49.5
Districts	Awabel	343	76.56	105	23.44
	Debay Tilat-gin	396	67.35	192	32.65
	Debre Ealias	291	49.24	300	50.76
	Debre Markos Town	57	11.38	444	88.62
	Dejen	168	27.18	450	72.82
	Enebsie Sar Midir	33	5.98	519	94.02
	Sinan	124	52.99	110	47.01
Agroecology	Highland and hot area	510	93.92	33	6.08
	Highland and cold area	110	47.01	124	52.99
	Low land area	393	70.05	168	29.95
	Mid land area	1107	50.46	1087	49.54
Climate	Dega	124	52.99	110	47.01
	Kolla	168	30.43	384	69.57
	Woina-daga	1120	40.79	1626	59.21
Household head	Female	228	35.29	418	64.71
	Male	1184	41.03	1702	58.97
Food expense	< 2100	944	36.24	1661	63.76
	≥ 2100	468	50.49	459	49.51
COVID-19	No	121	8.6	242	11.4
	Yes	370	26.2	1118	52.7
Price inflation	No	116	8.2	127	6.0
	Yes	677	47.9	1570	74.1
Conflict	No	32	2.3	149	7.0
	Yes	120	8.5	137	6.5
Weather change	No	141	10.0	108	5.1
	Yes	142	10.1	87	4.1

### Associated factors of household non-resilience to food insecurity

From the findings of this study, marital status, occupation of the household head, monthly income, residence district, agro-ecological condition, climate, effects of COVID-19, and price inflation were the most important factors contributing to food insecurity (Table 5).

**Table 5:** Associated factors of Household non-resilience to food insecurity at the time of COVID-19 pandemic and public emergency, Northwest Ethiopia, 2020.

Variables	Characteristics	Resilience			
		No	Yes	CoR at 95% CI	AoR at 95%CI
Marital status	Divorced	205	70	2.08 (1.57, 2.76)	**2.54 (1.65, 3.87)**
	Married	1284	912	1	1
	Single	479	316	1.08 (0.91, 1.27)	0.61 (0.47, 0.78)
	Widowed	152	114	0.95 (0.73, 1.23)	1.24 (0.81, 1.89)
Residence	Rural	1082	1316	1	1
	Urban	1038	96	13.2 (10.51, 16.45)	1.04 (0.34, 3.16)
Paternal education	Cannot read and write	668	893	1	1
	Can read and write	378	378	1.34 (1.12, 1.59)	0.6 (0.47, 0.78)
	College and above	429	42	13.66 (9.79, 19.04)	0.87 (0.46, 1.65)
	Primary (grades 1–8)	408	69	7.91 (6.01, 10.40)	2.73 (0.87, 3.98)
	Secondary (grades 9–12)	237	30	10.56 (7.13, 15.64)	2.3 (0.39, 3.80)
Maternal education	Cannot read and write	976	1193	1	1
	Can read and write	428	131	3.99 (3.23, 4.94)	2.15 (0.59, 2.19)
	College and above	234	12	23.8 (13.26, 42.84)	2.91 (0.33, 6.37)
	Primary (grades 1–8)	285	55	6.33 (4.69, 8.56)	0.88 (0.57, 1.34)
	Secondary (grades 9–12)	197	21	11.46 (7.26, 18.12)	0.9 (0.50, 1.63)
Occupation	Daily labourer	133	12	1.39 (0.73, 2.64)	**2.37 (1.15, 4.87)**
	Farmer	920	1288	0.09 (0.07, 0.12)	0.13 (0.08, 0.21)
	Government employee	375	27	1.74 (1.10, 2.77)	**2.06 (1.05, 4.05)**
	Housewife	150	17	1.11 (0.63, 1.94)	1.55 (0.08, 3.00)
	Merchant	542	68	1	1
Family size	< Five	1863	976	1.17 (1.00, 1.35)	2.67 (0.11, 3.39)
	≥ Five	257	436	1	1
Monthly income	≤ 2100	1661	944	1.79 (1.54, 2.09)	**1.34 (1.05, 1.70)**
	> 2100	459	468	1	1
Districts	Awabel	105	343	1	1
	Debay Tilat-gin	192	396	1.58 (1.02, 2.09)	**1.44 (0.99, 2.07)**
	Debre Ealias	300	291	3.37 (2.57, 4.42)	**3.6 (2.31, 5.61)**
	Debre Markos Town	444	57	25.4 (18.90, 18)	3.50 (0.96, 12.52)
	Dejen	168	450	8.75 (6.60, 11.59)	**4.21 (3.56, 6.78)**
	Enebsie Sar Midir	519	33	51.6 (33.96,77.74)	**31.3 (15.65, 48.15)**
	Sinan	110	124	2.89 (2.07, 4.06)	**1.86 (1.20, 2.89)**
Agro-ecology	Highland and hot area	510	33	15.2(10.57, 21.79)	**11.5 (5.37, 16.77)**
	Highland and cold area	110	124	0.87 (0.67, 1.14)	0.42 (0.12, 1.05)
	Low land area	393	168	2.3 (1.88, 2.80)	**1.35 (1.02, 2.56)**
	Mid land area	1107	1087	1	1
Climate	Dega	110	124	1	1
	Kolla	384	168	2.56 (1.88, 3.53)	**2.17 (1.02, 3.15)**
	Woina-daga	1626	1120	1.64 (1.25, 2.14)	**1.06 (1.00, 1.89)**
Household head	Female	418	228	1.25 (1.07, 1.52)	1.08 (0.76, 1.52)
	Male	1702	1184	1	1
Fear of COVID-19	No	242	121	1	1
	Yes	1118	370	1.51 (1.18, 1.94)	**1.23 (1.02, 1.50)**
Frustration with price inflation	No	127	116	1	1
	Yes	1570	677	2.13 (1.62, 2.77)	**1.89 (1.42, 2.56)**
Conflict	No	149	32	1	
	Yes	137	120	(0.16, 1.09)	0.02 (0.01, 1.03)

Significant values are in bold.

### Spatial distribution of household non-resilience to food insecurity

**Figure 3:**
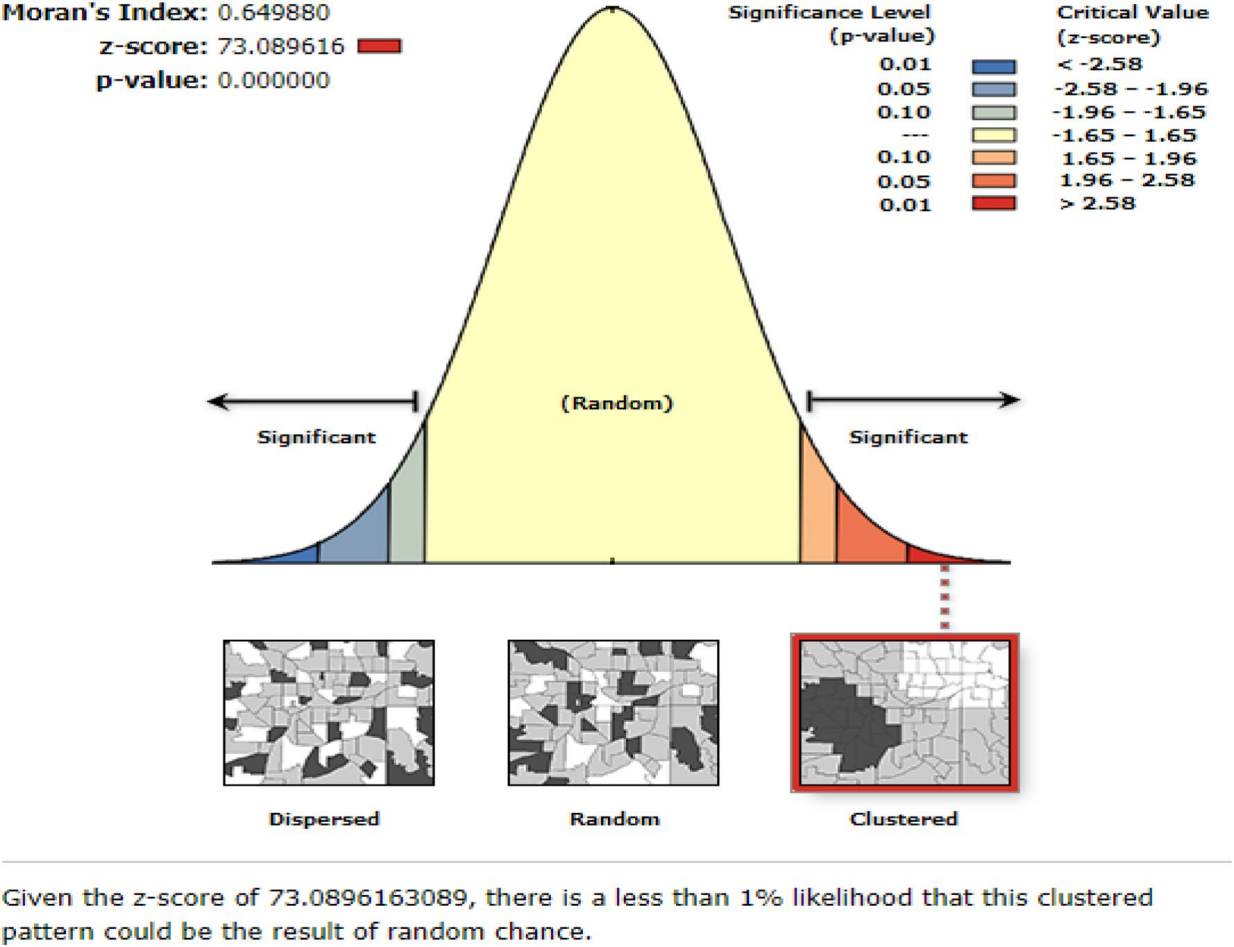
A global spatial autocorrelation analysis to explore the spatial pattern non-resilience to food insecurity during COVID-19 pandemic in East Gojjam Zone, Northwest Ethiopia, 2021.

**Figure 4:**
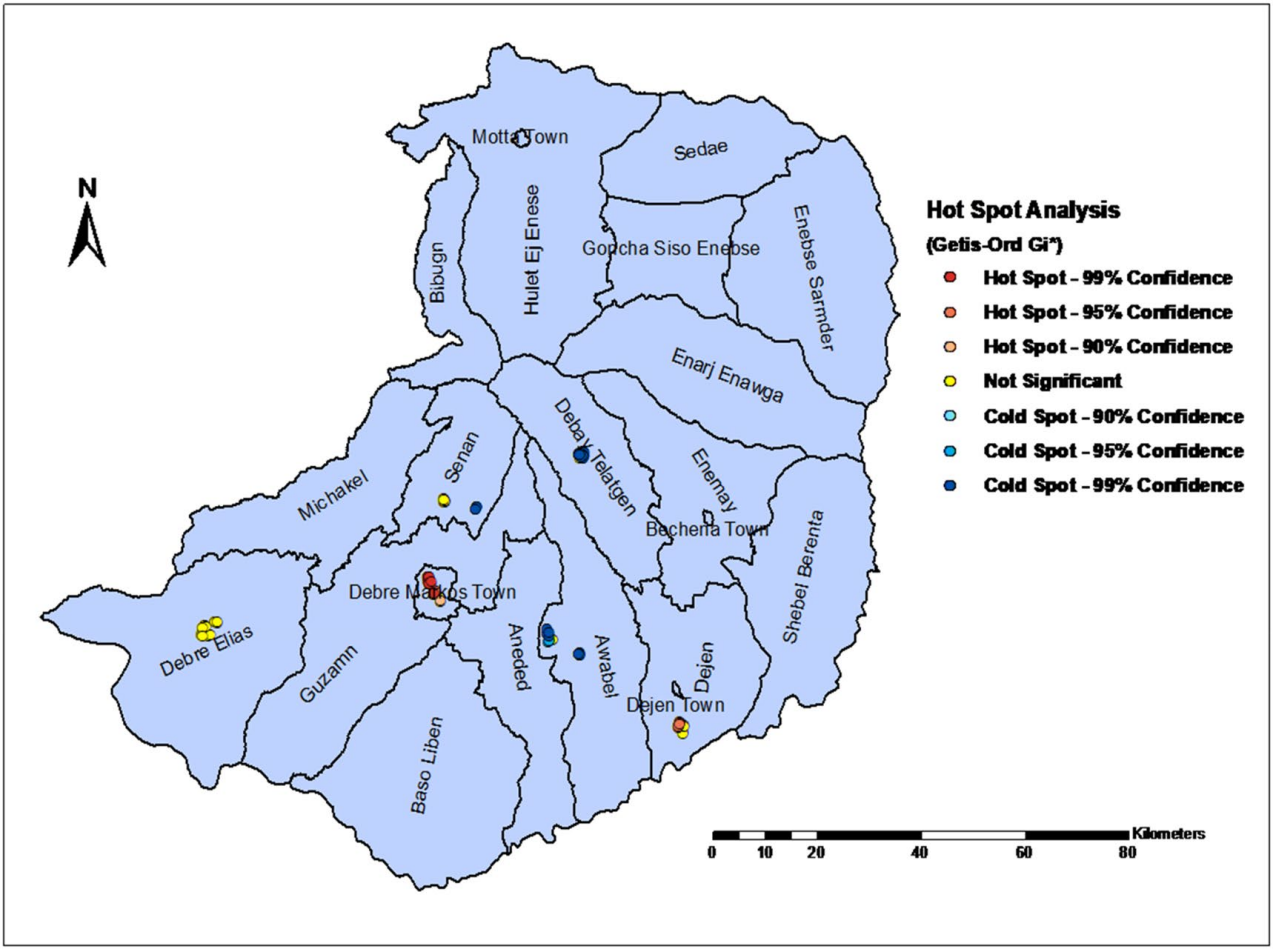
Hotspot areas of household non-resilience to food insecurity during COVID-19 pandemic in East Gojjam Zone, Northwest Ethiopia, 2021.

In the GSA analysis, the Moran's I = 0.65 and p-value0.001 indicated a positive autocorrelation, i.e. a clustered pattern of non-resilience to food insecurity across the entire study areas (Fig. 3). This result suggests that additional hotspot analysis be performed to identify local clusters (Fig. 4).

### Hotspot analysis

The hotspot analysis with Getis-Ord Gi* statistic directed the clusters of household non-resilience to food security at Debre Markos town and Kurar kebele from Dejen district, as shown in Fig. 4. (Both at 95% and 99% confidence interval).

### Coping strategies of non-resilience during COVID-19 pandemic and public emergency

This study found that during the COVID-19 pandemic and public emergency, more than two-thirds of households did not consider any of the coping strategies to be resilient. As a coping strategy, nearly 17.5% limited their meal preparations per week. During the COVID-19 pandemic and public emergency, nearly 15% of households used daily food restriction as non-resilience coping strategy (Table 6).

**Table 6:** Coping strategies of non-resilience during COVID-19 pandemic and public emergency, Northwest Ethiopia, 2021.

Variables	Characteristics	No	%
Relay on less preferred food items	Every day	232	6.8%
	Once a week	161	4.6
	None	2917	82.6
	2–3 times/week	116	3.3
	4–6 times/week	106	3.0
Food borrowing	Every day	227	6.4
	Once a week	388	11.0
	None	2608	73.8
	2–3 times/week	179	5.1
	4–6 times/week	130	3.7
Purchasing food on credit	Every day	144	4.1
	Once a week	300	8.5
	None	2774	78.5
	2–3 times/week	188	5.3
	4–6 times/week	126	3.6
Hunting wild animals	Every day	41	1.1
	Once a week	20	0.6
	None	3441	97.4
	2–3 times/week	11	0.3
	4–6 times/week	19	0.5
Consuming seed stocks	Every day	93	2.6
	Once a week	174	4.9
	None	3192	90.4
	2–3 times/week	41	1.2
	4–6 times/week	32	0.9
Sending children for beg	Every day	62	1.8
	Once a week	86	2.5
	None	3434	97.2
	2–3 times/week	6	0.2
	4–6 times/week	6	0.2
Sending children elsewhere to eat	Every day	42	1.2
	Once a week	320	9.1
	None	3050	86.4
	2–3 times/week	96	2.7
	4–6 times/week	24	0.7
Limiting meal proportions	Every day	201	5.7
	Once a week	617	17.5
	None	2186	61.9
	2–3 times/week	366	10.4
	4–6 times/week	162	4.6
Restricting adult’s food consumption	Every day	478	13.6
	Once a week	87	2.5
	None	2742	77.6
	2–3 times/week	73	2.1
	4–6 times/week	152	4.3
Feeding more the working group of the family	Every day	298	8.5
	Once a week	97	2.7
	None	2830	80.1
	2–3 times/week	114	3.2
	4–6 times/week	193	5.5
Reducing meal number	Every day	233	6.7
	Once a week	377	10.7
	None	2387	67.6
	2–3 times/week	350	9.9
	4–6 times/week	184	5.2
Skipping meals in a day	Every day	58	1.6
	Once a week	163	4.6
	None	3254	92.1
	2–3 times/week	39	1.1
	4–6 times/week	18	0.5
Harvesting immature crops	Every day	59	1.6
	Once a week	214	6.1
	None	3154	89.3
	2–3 times/week	68	1.9
	4–6 times/week	37	1.0

### Challenges and opportunities to exit from household non-resilience to food security

As of the respective bodies addressed, the challenges explained in the community were the degree of poverty, the COVID-19 pandemic, public emergency and lockdown, limited variety, and decreased crop productivity. On the other hand, there were opportunities to mitigate the deteriorating effects of household food insecurity, such as increased seed production for farmers and distribution of emergency food as early management of food insecurity (Debre Markos Social and Labor service department, Child and Women Department, Zonal COVID-19 prevention committee leader, and Zonal agriculture and seed lab department). The poorest of the poor households were the most difficult to break free from non-resilience, and they require special attention in future interventional strategies.

“…During this COVID 19 pandemic and public emergency, there are some challenges to address food insecurity among the vulnerable groups, especially in the kebele levels, who are the poorest of poor, and which sections of the community are most vulnerable to food insecurity…” (DM Social and Labor Service Department, 2020, Women and Child Department Office, 2020).

The COVID-19 pandemic is currently a challenge for developing countries due to international lockdown and movement restrictions, which have indirectly affected the movement of international aiding agencies. They also reported that some parts of Ethiopia including the study area,, particularly the Abay Gorge areas and hilly and mountainous areas, are severely affected by the issue of food insecurity.

“…COVID 19 poses a significant food security problem compared to other hazards, but the problem caused by COVID 19 varies from urban to rural. It is not known to be widespread in rural areas and is not yet available, but I think it could be more dangerous in the future, but in the city, it has spread as I said before…” (COVID-19 prevention Committee Leader, 2020).

The key to overcoming such issues is to put in more effort on the community, such as by cultivating new seeds and increasing productivity. Especially in this society, which consumes the majority of grains such as barley, wheat, corn, and sorghum, and has little chance of producing other types of products such as fruits and vegetables.

“…Now, in our office, we need to control seed quality to solve the food security problem, and we will have more seed multiplication and new seeds to be imported and preserved in quality. Like mangoes and avocados, our seeds are our source of quality and health, and they can be produced in large quantities and distributed to the poorest community at an affordable price. However, due to the lack of quality and quantity of the produce of products, if we take Orange as an example, it costs 50 birr per kilo. Therefore, food security can be addressed by increasing production and productivity…” (Zonal Agriculture and Seed Lab Department, 2020).

An alternative to addressing the issue of food security is for the state government to increase its productivity by inspecting the lands occupied by investors and transferring them to a development individual or organization for development. Similarly, there has been a significant increase in seed production this year to address such issues and ensure food security.

“…Yes of course because the government has made relentless efforts to address food insecurity by asking for assistance from top to bottom. After assessing and selecting risk takers the government distributes food and other materials given from internal and external funding in order to prevent this pandemic…” (Disaster Prevention and Preparedness Department, 2020).

## Discussion

A number of household food insecurity studies^47–56^ were conducted in Ethiopia, demonstrating the alarming scale of food insecurity and its deteriorating effects on the population's health and socioeconomic status due to various shock exposures. As a result, the purpose of this study was to investigate households' resilience to food insecurity during the COVID-19 pandemic shock and public emergency declared by the Ethiopian government.

According to the findings of this study, nearly two-thirds of the households (60.02%, 95% CI 58.40, 61.64) were non-resilient due to household food insecurity during the COVID-19 pandemic and public emergency. This finding was consistent with Tehran-Iran studies^57^ and it revealed that it was nearly doubled compared to a previous study conducted before the pandemic across Northeast and North African countries^58^. This could be because, in addition to geographical, social, cultural, and economic differences, the COVID-19 pandemic and public emergency exacerbated the problem of household non-resilience in the East Gojjam districts. The social and economic fallout magnified the public health crisis: job losses, school closures, and increased poverty. These consequences are borne primarily by vulnerable populations around the world, including those in Africa. The pandemic could undermine hard-won development gains and jeopardize household resilience to food insecurity, as well as have a more practical impact on international organizations working on household resilience to food insecurity^41,59^. This finding was also lower than among a multi-country cross-sectional survey conducted across nine African countries (Chad, Djibouti, Ethiopia, Kenya, Malawi, Mali, Nigeria, South Africa, and Uganda)^26^, and one of the pocket study conducted in Addis Ababa town administration^5^. This is due to the fact that the COVID-19 pandemic and public emergency primarily affected urban settings and were more vulnerable for non-resilience to household food insecurity than the rural settings^38,60^. However, when compared to other similar studies, the current study included different agro-ecological areas, as well as urban and rural settings, which directly affects the prevalence of non-resilience of household food insecurity. Divorce was associated with non-resilience to food insecurity during the COVID-19 pandemic and public emergency, especially for women-headed households, according to this study. This could be explained by the fact that married people are expected to have a stable family life, which may have a positive impact on household food security, whereas divorcees may face several socioeconomic challenges because their previous household assets and other properties could be divided or lost following the divorce^61^. As a result, special consideration must be given to this segment of the population in order to communicate with and capacitate them in terms of responding to household non-resilience to food insecurity during this time of COVID-19 pandemic and public emergency. Similarly, monthly income was found to be one of the main associated factors with households' non-resilience to food insecurity during the COVID-19 pandemic shock. This could be attributed to the pandemic and public emergency, which had an impact on the labor market and reduced household income, particularly in urban areas^62,63^. Moreover, during lockdown periods and public movement restrictions during the COVID-19 pandemic, means of earning income were reduced, limiting households' purchasing power of food consumptions, which is one of the resilient mechanisms against transient food insecurity. Data published elsewhere show that low-income households and those reliant on labor-intensive income earners were more vulnerable to income shock and consumed less food during the COVID-19 pandemic^64–66^. This implies that during times of public emergency such as COVID-19, decision-makers, policymakers, and others should place special emphasis on households that rely on labor-intensive jobs.

Furthermore, market inflation was strongly linked to household vulnerability in the face of food insecurity. This could be due to deterioration in the food value chain, hoarding by local market dealers, and panic buying by the community at the start of the COVID-19 pandemic, resulting in a supply shortage to the market^61,63,67^. This implies that the national government, local governments, communities, as well as other concerned bodies should collaborate, design and implement rigorous mechanisms to reduce price inflation during the COVID-19 pandemic and public emergency, specifically setting a fixed price for foods and commodities for consumption.

The most hotspot areas identified in the agro-ecological analysis of household food insecurity resilience were Debre Markos town and Kurar kebele (in the Abay valley with lowland and hot weather conditions), which was consistent with Alemu and his colleagues' previous food insecurity level study^56^. During the COVID-19 pandemic and public emergency, transportation from town to town, even across borders, was restricted, affecting the resilience status of households. Price inflation in Debre Markos, in particular, may affect households' purchasing power of basic food commodities. Prior to the occurrence of the COVID-19 pandemic and public emergency, the lowland areas, particularly the kebeles settled in the Abay Gorge, were identified as the hottest spot areas affected by household food insecurity, which could be exacerbated by the pandemic^23,56^.

In terms of household occupation, daily laborers and government employees were more affected by household food insecurity and were less resilient. It was the fact that, during the COVID-19 pandemic and public emergency, the government-imposed lockdown and movement restrictions had resulted in a loss of income, particularly for individuals whose lives were dependent on daily wages. Similarly, in Ethiopia, the lives of government employees are entirely dependent on the products of rural farmers. Farmers' availability of basic foods and commodities to urban communities, including government employees, was hampered by movement restrictions^61^.

In this study, more than two-thirds of the households chose no coping strategies to deal with the occurrence of food insecurity. Less than one-fifth of the households relied on food consumption changes/adjustments, primarily limiting food consumption and using less preferred or low-quality foods, as these strategies are easily adjustable. However, these coping strategies are harmful to people's health and which will affect the capacity of resilience negatively^68^. The main reason for households' reliance on coping strategies for food consumption adjustments was associated with the level of asset ownership, which was discovered to be the main determinant factor of food insecurity resilience. The poor chose food consumption strategies to cope with the shocking exposure (in this case, the COVID-19 pandemic) because the poor are usually affected by shocks^69,70^.

The challenges posed by the COVID-19 pandemic on household resilience to food insecurity in East Gojjam Zone, as elaborated by responsible government and non-governmental bodies, were the degree of poverty at the household level, the government's declaration of public emergency and lockdown, limited variety and decreased productivity of farmers' crops. While there were opportunities in disguise that could help to address the shadow of household food insecurity, such as increased seed production to distribute to farmers and emergency food distribution as early management of food insecurity (Debre Markos Social and Labor service department, Child and Women Department, Zonal COVID-19 prevention committee leader, and Zonal agriculture and seed lab department).

## Theoretical and practical implication of this study

It is real that non-resilient households for food insecurity are more vulnerable to social, political, and economic crises, necessitating a comprehensive package of assistance from the federal government and international relief organizations to alleviate acute household food shortages. Furthermore, the households' coping strategies identified during this pandemic and public emergency must be strengthened and incorporated as core mitigation strategies of household non-resilience to food insecurity for the study area and similar settings across the country. Furthermore, this study found that urban households and households in the low-land areas of the Nile gorge were hotspot areas and more prone to non-resilience to food insecurity, which deserves more attention and requires integrated services that can minimize the cost of living, mitigates repeated drought attacks among Nile gorge households, and other COVID-19 induced non-resilience mitigation strategies.

## Strength and limitation of this study

The strength of this study was that it used mixed methods research to answer the various research questions in a comprehensive manner and attempted to provide the prevalence of household non-resilience to food insecurity, identified the associated factors, hotspot areas, household coping strategies used during the pandemic, and the opportunities and challenges to exit from this critical issue in a single article for the scientific community. It also used electronic assisted and GPS linked data collection techniques, both of which were critical for maintaining data quality throughout the data collection period. Another advantage of this study was that it included both urban and rural households by taking into account the different agro-ecologies classified in previous studies^43^. This study looked at hilly and mountainous areas in cold and hot weather, which are both more vulnerable to soil erosion and drought, respectively, and bear the triple burden of household non-resilience to food insecurity, in addition to the COVID-19 pandemic and public emergency. This study also included low-land areas with hot weather, as well as mid-land areas with brown, black, and red soil types, all of which are important for household resilience to food security in both rural and surrounding urban populations. Furthermore, this study also estimated the prevalence of household non-resilience to food insecurity by considering the large and representative sample size and by using the internationally standardized tool that is the Resilience Index Measurement and Analysis II (RIMA-II) validated by the United Nations Food and Agricultural Organization (FAO)^12^. However, this study may be limited by the social desirability bias because households during the COVID-19 pandemic and public emergency may believe that the government will provide assistance while undermining their income and other resources critical for household resilience to food security.

## Conclusion and recommendations

In terms of spatial heterogeneity, the prevalence of non-resilience to food insecurity was found to be higher prior to the era of the COVID-19 pandemic and public emergency, which is a concerning issue in the study area and at the national level in general. Daily laborers and government employers, kola and Woyna-Dega climatic zones, highland and low land with hot areas, household monthly income, market price inflation, and being a female-headed household were significant factors associated with household non-resilience to food insecurity. As a result, there is a need to control market inflation for basic food commodities based on baseline research, as well as update international humanitarian agencies to provide food and consumables access in hotspot areas. The respective bodies should provide assistance and advice on agro-ecologically adapted and productive agriculture extension services. Female-headed households should be given assistance to increase their decision-making power in order to improve their resilience capacity. Moreover, People's food eating patterns changed as a result of the coping mechanisms employed by households to fortify themselves against the startling COVID-19 pandemic exposure, which negatively impacted their health and reduced their resilience as they have a bi-directional influence. As a result, strategies tailored to minimize this issue should be considered and designed with the local context in mind.

## Supplementary Information

Supplementary Information.

## Data Availability

All data generated or analysed during this study are included in this published article (and its Supplementary Information files).
